# Supplementary material for: Protective Mechanism of Rosa roxburghii Tratt Fermentation Broth against Ultraviolet-A-Induced Photoaging of Human Embryonic Skin Fibroblasts
Source: Antioxidants (Basel). 2024 Mar 21;13(3):382. doi: 10.3390/antiox13030382 (PMC10968601; doi:10.3390/antiox13030382)
Supplement: Supplementary file 1 [file antioxidants-13-00382-s001.zip › antioxidants-2847288-supplementary.pdf]

Supplementary material

Table S1: Primer sequences for RT-qPCR

Gene	Primer
actin	F: GCG AGA AGA TGA CCCAGAT<br>R: ATC ACG ATG CCA GTGGTA
TGF-β	F: CAACCACCAGGGCATCCA<br>R: TCGTGGTCCCAGCACTCA
TIMP	F: CTTTTCAGAGCCTTGGAGGAG<br>R: TGGGGACACCAGAAGTCAAC
p38	F:TTAACAGGATGCCAAGCCATGA<br>R:GGCACCAATAAATACATTCGCAAAG
JNK	F:CTGTGTGGAATCAAGCACCTTCA<br>R:CTGGCCAGACCGAAGTCAAGA
ERK2	F: 5’-TGTTCCCAAATGCTGACTCCAA-3’<br>R:5'-TCGGGTCGTAATACTGCTCCAGATA-3’
c-Jun	F: AAT AAC ACA GAG AGA CAG ACT TG<br>R: CTT GGA TAC CCT TGG CTT TAG
c-Fos	F: GTG TGT ATT GTT CCC AGT GA<br>R: AGTTAATGC TAT GAG AAG ACT AAG G
Smad3	F: GGCTGCTCT CCA ATG TCA<br>R: CAC TCTGCGAAG ACC TCC
Smad7	F: TTC CCT CCA AGAAGGATT TG<br>R: ACG AGT GTA TGA GTTGTAGAA G
MMP-1	F: GCA TATCGA TGC TGC TCT TTC<br>R: GAT AAC CTGGAT CCA TAG ATC GTT
MMP-2	F: GGA GGA GAAGGCTGT GTT<br>R: TAA AGG CGG CAT CCACTC
MMP-3	F: CAA AAC ATA TTT CTT TGT AGAGGACAA<br>R: TTC AGC TAT TTG CTT GGGAAA
MMP-9	F: ATG TAC CCT ATG TAC CGC TTC<br>R: GTG TGG TGG TGG TTG GAG
Nrf2	F: CAACTCAGCACCTTGTATC<br>R: TTCTTAGTATCTGGCTTCTT
HO1	F: CAAGCGCTATGTTCAGCGAC<br>R: GCTTGAACTTGGTGGCACTG
CAT	F: CCTTCGACCCAAGCAA<br>R : CGATGGCGGTGAGTGT
SOD	F:TGGAGATAATACAGCAGGCT<br>R: AGTCACATTGCCCAAGTCTC

GSH-Px	F: AGAAGTGCGAGGTGAACGGT<br>R: CCCACCAGGAACTTCTCAAA
COL I:	F: GTGCTAAAGGTGCCAATGGT<br>R:GTGGGGAATGGCAAGCAAAA
COL III:	F: CCAGGAGCTAACGGTCTCAG<br>R: CAGGGTTTCCATCTCTTCCA

Table S2: Summary of differentially expressed genes

group	differentially expressed genes	differentially up-regulated genes	differentially down-regulated genes
Control_vs_Model	1117	465	652
Control_vs_RRT	1060	326	734
Model_vs_RRT	1933	847	1086

Table S3: Top 20 summary table of GO annotation analysis results

Term description	Term type	DEG_1 num
immune system process	biological_process	207
reproductive process	biological_process	201
multicellular organismal process	biological_process	385
localization	biological_process	429
developmental process	biological_process	621
response to stimulus	biological_process	720
metabolic process	biological_process	851
biological regulation	biological_process	1360
cellular process	biological_process	1639
cellular component organization or biogenesis	biological_process	694
extracellular region part	cellular_component	266
membrane	cellular_component	644
protein-containing complex	cellular_component	720
membrane part	cellular_component	750
organelle part	cellular_component	1090
organelle	cellular_component	1269
cell part	cellular_component	1925
molecular function regulator	molecular_function	242
catalytic activity	molecular_function	732
binding	molecular_function	1720

Table S4: Top 20 summary table of KEGG annotation analysis results

First category	Second category	Number of genes
Cellular Processes	Cell growth and death	26
	Cell motility	128
Environmental Information Processing	Signaling molecules and interaction	107
	Signal transduction	241
	Membrane transport	10
Genetic Information Processing	Translation	28
	Transcription	21
	Replication and repair	64
	Folding, sorting and degradation	36
Metabolism	Xenobiotics biodegradation and metabolism	27
	Nucleotide metabolism	30
	Metabolism of terpenoids and polyketides	3
	Metabolism of other amino acids	15
	Metabolism of cofactors and vitamins	25
	Lipid metabolism	39
	Glycan biosynthesis and metabolism	25
	Energy metabolism	11
	Carbohydrate metabolism	43
	Biosynthesis of other secondary metabolites	2
	Amino acid metabolism	35

Table S5: Summary of photoaging-related pathway genes

Gene Num	Description	Pvalue	Padjust
27	DNA replication	8.63E-21	2.79E-18
45	Cell cycle	1.38E-14	2.22E-12
26	p53 signaling pathway	5.05E-09	2.72E-07
41	Calcium signaling pathway	7.68E-06	0.000225424
24	ECM-receptor interaction	7.07E-06	0.000228256
83	Pathways in cancer	0.000211641	0.004021175
32	Cellular senescence	0.001332357	0.017214056
55	PI3K-Akt signaling pathway	0.001605322	0.019943042
26	Apoptosis	0.001897709	0.022702227
22	TNF signaling pathway	0.004039141	0.03953462
17	AGE-RAGE signaling	0.034559088	0.186043091

	pathway in diabetic complications		
41	MAPK signaling pathway	0.036046883	0.1908712
16	TGF-beta signaling pathway	0.03929143	0.198298934
